# Self-perception of dual career barriers and athletic identity in student-athletes with disabilities according to disability type and level of professionalization

**DOI:** 10.1038/s41598-023-47881-4

**Published:** 2023-11-22

**Authors:** M. J. Maciá-Andreu, R. Vaquero-Cristóbal, L. Meroño, L. Abenza-Cano, J. A. García-Roca, F. J. Cánovas-Álvarez, A. Díaz-Aroca, L. Capranica, M. Stanescu, A. Pereira, M. Doupona, F. Mendes, A. Figueiredo, E. Isidori, A. Sánchez-Pato, A. Leiva-Arcas

**Affiliations:** 1 Facultad de Deporte, Universidad Católica San Antonio de Murcia, 30107 Murcia, Spain; 2 Departament of Physical Activity and Sport Sciences, Faculty of Sport Sciences, University of Murcia, 30720 San Javier, Spain; 3 Centre for Olympic Studies, Universidad Católica San Antonio de Murcia, 30107 Murcia, Spain; 4 European Athlete as Student Network, Ghaxaq, 1025 Malta; 5 Department of Movement, Human and Health Sciences, Università Degli Studi di Roma Foro Italico, 00135 Rome, Italy; 6 National University of Physical Education and Sport of Bucharest, 060057 Bucharest, Romania; 7 Department of Sport Sciences and Motricity, Polytechnic Institute of Viseu, 3504-510 Viseu, Portugal; 8 University of Ljubljana, 1000 Ljubljana, Slovenia; 9 Facultad de Ciencias de la Salud, Universidad Internacional de la Rioja, 26006 Logroño, Spain

**Keywords:** Health care, Psychology

## Abstract

The objective of this study was to analyze the perceived barriers to dual career success and athletic identity of student-athletes according to disability type and level of professionalization. The final sample consisted of 203 student-athletes with disabilities from five European countries. The questionnaires used were ESTPORT, EBBS and AIMS. Depending on disability type, it was found that student-athletes with hearing and physical impairment showed the highest difficulty in reconciling sports and studies (p = 0.001); that student-athletes with a hearing impairment showed the highest score in the barrier ‘the cost of education is high’ (p = 0.023); that student-athletes with a physical impairment had the highest scores in the barrier ‘Exercise tires me’ (p = 0.013); that student-athletes with cerebral palsy showed the highest scores in the barrier ‘I do not have enough university/educational institution support’ (p = 0.014) and ‘Exercise facilities do not have convenient timetables for me’ (p = 0.001). Depending on sports professionalization level, semi-professional student-athletes showed the highest values in the barrier ‘the university/educational institution is far from my training center’ (p = 0.040); while professional student-athletes had the highest score in the barrier ‘exercise takes too much time from family responsibilities’ (p = 0.034). In most of the variables related to identity as athletes, professional student-athletes showed the highest values, followed by semi-professional athletes (p = 0.043- < 0.001). In conclusion, the self-perception of barriers is quite relevant, with differences arising from disability type and level of professionalization, whereas the identity as an athlete is only different according to the level of professionalization.

## Introduction

The dual career has been defined as the process of combining a sports career with an academic career^1^. It seems that pursuing a dual career is usually a challenging balancing act for young student-athletes^2^, mainly because both sports and studies are two time-consuming activities^3^, with this combination posing a number of difficulties in their attempt to reconcile the academic and sports training process^4^, such as academic dropout, withdrawal from the sports activity, the process of integration into professional activity, in relation to the emotional and financial aspects involved^5,6^, and relationship issues, with a greater proportion of time allocated to sports training at the expense of time for study, family and friends^7^. However, dual careers can also have a positive impact on athletic identity^8^. Athletes who also study can develop a multidimensional identity that enriches them as individuals and enables them to better cope with their post-sports life^9^. Combining high-level sports with higher education helps to create a less rigid personality that allows them to plan their future with greater freedom^10^.

In recent years, there has been a growing interest, particularly from public authorities in the European Union, in developing initiatives, strategies and policies that promote dual careers through direct grants and support for research in this field^11^*.* In 2012, the European Commission published a set of guidelines for Member States to promote national policies on dual careers in the high-performance sector^12^. The aim of these proposals is to ensure that European athletes can achieve success in the dual career, being able to successfully address both the sporting and academic spheres, to be as well prepared as possible for employment after their sporting careers have come to an end^13^, a reason why most student-athletes embark on dual careers^14^. But this is not easy. As previous studies have shown, student-athletes, regardless of their level of performance, spend a significant amount of time on sports training, to the detriment of education^3,15^ and it seems to be that those who are not prepared for a post-sports life^16,17^, have high rates of depression or anxiety^18^, as well as financial problems^13^.

The dual career support system proposed in the European Union, where dual careers are a priority in transnational policies^11^, differs from that in other reference countries in dual careers, such as the United States, where the responsibility for supporting the dual career of student-athletes is the responsibility of individual university initiatives, which seek to strengthen their corporate reputation^19^. However, it is also true that this model is favored at the state policy level, as high-level sports in the United States relies heavily on private investment in university sports^20^.

As a result, in recent years, scientific achievements in this field have increased considerably, the horizon of knowledge has broadened significantly^21^, and the dual career has been described as an emerging research area^21^. Most of these studies have aimed to connect, at the European level, both sports and education systems, which are still strongly disconnected in this territory^22^. More specifically, studies have focused on student-athlete’s analyzing their experiences, perceptions on athlete identity, career transitions, motivations, achievements, and reasons for dropout^17,21,23–32^; the effectiveness of practices in sports and educational environments to achieve a successful dual career, comparing different countries/models, career assistance programs, educational mobility, or talent development programs, among others^1,21,33,34^; or global aspects related to organizational and governmental policies on dual career and the management of top-level sport across Europe^4,35–37^.

However, almost all of these studies have focused on non-disabled student-athletes, while disabled student-athletes have been completely neglected so far. Only a few research studies have focused on the dual career of disabled student-athletes^38–40^, despite the growing importance given to the dual career of the student-athlete in recent years, and the fact that the Council of Europe establishes the protection of student-athletes with disabilities as one of the main challenges in interventions^39,41^. Because of the above, there is a lack of knowledge about the dual career in disabled student-athletes, although this is a particularly sensitive population, given that in addition to the usual difficulties experienced by dual career student-athletes, they often have difficulties in normalized inclusion in the system^38,40^, which is reflected in the barriers they face to ensure success in the sport and educational context. Preliminary studies have found that barriers may be accentuated by the additional limitations they encounter in both the educational^40^ and sporting spheres^42^, self-perceiving barriers to dual careers to a greater extent than non-disabled peers, especially in reference to time management capacities and lack of flexibility in this area, institutional support, or moving from one location to another^39^. This is reflected on the fact that the limited data available indicate that people with disabilities participate in sport activities and attend university courses significantly less than their non-disabled peers^43^, and that sport policies aimed at developing the career paths of disabled athletes are significantly less advanced than those of non-disabled athletes^44^.

In this difficult context, one of the keys for student-athletes with disabilities to not abandon their sporting career, especially at an early age and in the non-professional sphere, is the perception they have on their identity as an athlete^45,46^. This type of identity is understood as the self-perception of an individual based on the link that he/she has created with the sport that he/she has played for a large part of his/her life, and the degree of importance of this dimension with respect to the rest of the areas of life^47^. The connection between athlete identity and dual career success has been extensively studied, with contradictory results. Thus, while some studies have found that a strong perception of identity as an athlete may be related to lower academic performance^48^, other studies have found that student-athletes' commitment to both athletics and academics plays an important role in their academic performance^49^. However, despite its importance, this parameter has not been addressed in previous research on dual career athletes with disabilities. Therefore, research is needed to address this question in order to see how the relationship between these variables behaves in disabled athletes.

In addition, all the studies that have analyzed the dual career success of student-athletes with disabilities have analyzed this group as a whole. But previous studies have found that academic career success may depend on the type of disability of the individual^50–52^. Therefore, an analysis of the barriers to success in dual careers according to disability is necessary to develop specific policies and programs for the most vulnerable groups. Similarly, in non-disabled student-athletes, it has been found that the level of sport professionalization could have an influence on the perception of difficulties in finding a balance in the dual career^15^, but no studies have analyzed whether the level of sport professionalization could be a significant modulator for disabled student-athletes. Therefore, the objective of this research was to analyze the differences in the dual career of European student-athletes with disabilities, concerning dual career and exercise barriers and athletic identity, depending on disability type and level of sports professionalization.

Based on previous scientific literature, the research hypotheses posed are that there would be differences in the difficulty in achieving success in the dual career, as well as in the perception of barriers, depending on the type of disability and the competitive level. It was also hypothesized that the competitive level could be a determining factor in the participants' perception of identity as an athlete. However, the lack of previous studies on the issue of athlete identity according to the type of disability did not allow us to establish a clear hypothesis on whether there would be differences in this dimension.

## Methods

### Design

The study design was descriptive and cross-sectional, with non-probability convenience sampling. The STROBE statement^53^ was followed for the research design and development of the manuscript. Study participants gave their consent to participate prior to data collection and were informed of the research objectives and the confidentiality of the data obtained during the research. The institutional ethics committee reviewed and authorized the protocol designed for data collection (code: CE012101), in accordance with the code of the World Medical Association and the Declaration of Helsinki.

### Participants

The sample size was calculated using Rstudio 3.15.0 software (Rstudio Inc., USA). The significance value was set at α = 0.05. The standard deviation (SD) was established considering to perceived barriers of previous studies (SD = 0.75)^15^. With an estimated error (d) of 0.10, the required sample size for a 99% confidence interval (CI) was 200 subjects. The final sample consisted of 203 student-athletes with disabilities from five European countries (Spain, Portugal, Italy, Ireland, and Romania). The inclusion criteria were defined on the basis of previous studies^39^: (a) have a physical, sensory (visual or hearing) disability, or cerebral palsy; (b) been enrolled in a sports federation for at least three years; (c) to be currently enrolled in the last academic years of compulsory education (pre-university education), a university degree, a university master's degree, or a doctorate, and (d) have an adequate level of reading comprehension in English in order to complete the questionnaire.

The sample characteristics are shown in Table 1. Depending on the type of disability, significant differences were found only for level of professionalization. The visually impaired had a higher percentage of professional athletes, the hearing impaired had a higher percentage of semi-professional athletes, and the cerebral palsy group had a higher percentage of amateur athletes, while the physically impaired had the greatest heterogeneity in terms of level of professionalization (p = 0.007).

**Table 1:** Differences in socio-demographic; time distribution; studying and sporting characteristics depending on type of disability and level of sports professionalization.

Disability	Hearing (n = 34)	Visual (n = 44)	Physical (n = 87)	Cerebral palsy (n = 38)	Group differences	V
Age	23.00 ± 6.01	23.97 ± 7.51	26.02 ± 8.58	23.92 ± 7.41	F = 1.63, df = 3, p = 0.182	–
Gender	♂ 29 (58.8%)	♂ 30 (68.2%)	♂ 58 (66.7%	♂ 22 (57.9%)	χ^2^ = 1.61, df = 3, p = 0.656	–
	♀ 14 (41.2%)	♀ 14 (31.8%)	♀ 29 (33.3%)	♀ 16 (42.1%)		
Education	Pre: 15 (44.1%)	Pre: 19 (43.2%)	Pre: 20 (23.0%)	Pre: 13 (34.2%)	χ^2^ = 9.19, df = 6, p = 0.163	–
	UD: 15 (44.1%)	UD: 16 (36.4%)	UD: 49 (56.3%)	UD: 17 (44.7%)		
	Post: 4 (11.8%)	Post: 9 (20.5%)	Post: 18 (20.7%)	Post: 8 (21.1%)		
Professionalization	Amateur: 8 (23.5%)	Amateur: 9 (20.5%)	Amateur: 30 (34.5%)	Amateur: 21 (55.3%)	χ^2^ = 17.84, df = 6, p = 0.007**	V = 0.21
	Semi-prof.: 20 (58.8%)	Semi-prof.: 19 (43.2%)	Semi-prof.: 31 (35.6%)	Semi-prof.: 10 (26.3%)		
	Prof.: 6 (17.6%)	Prof.: 16 (36.4%)	Prof.: 26 (29.9%)	Prof.: 7 (18.4%)		
Stage	Beginning: 14 (41.2%)	Beginning: 26 (61.9%)	Beginning: 48 (55.2%)	Beginning: 21 (55.3%)	χ^2^ = 4.12, df = 6, p = 0.659	–
	Highest: 16 (47.1%)	Highest: 13 (31.0%)	Highest: 29 (33.3%)	Highest: 12 (31.6%)		
	End: 4 (11.8%)	End: 3 (7.1%)	End: 10 (11.5%)	End: 5 (13.2%)		
Hours/week study	31.12 ± 19.18	24.23 ± 14.16	25.00 ± 16.41	28.06 ± 15.54	F = 1.51, df = 3, p = 0.212	–
Hours/week sport	18.58 ± 38.24	13.09 ± 6.34	15.31 ± 9.04	12.52 ± 8.31	F = 0.92, df = 3, p = 0.427	–

Professionalization	Amateur (n = 68)	Semi-professional (n = 80)	Professional (n = 55)	Group differences	V/η^2^	
Age	24.25 ± 7.68	24.38 ± 7.35	25.63 ± 8.60	F = 0.57,df = 2, p = 0.566	–	
Gender	♂ 40 (58.8%)	♂ 55 (68.8%)	♂ 35 (63.6%)	χ^2^ = 1.57, df = 2, p = 0.454	–	
	♀ 28 (41.2%)	♀ 25 (31.3%)	♀ 20 (36.4%)			
Disability	H: 8 (11.8%)	H: 20 (25.0%)	H: 6 (10.9%)	χ^2^ = 17.84, df = 6, p = 0.007**	V = 0.21	
	V: 9 (13.2%)	V: 19 (23.8%)	V: 16 (29.1%)			
	Ph: 30 (44.1%)	Ph: 31 (38.8%)	Ph: 26 (47.3%)			
	CP: 21 (30.9%)	CP: 10 (12.5%)	CP: 7 (12.7%)			
Education	Pre: 24 (35.3%)	Pre: 27 (33.8%)	Pre: 16 (29.1%)	χ^2^ = 0.90, df = 4, p = 0.924	–	
	UD: 30 (44.1%)	UD: 39 (48.8%)	UD: 28 (50.9%)			
	Post: 14 (20.6%)	Post: 14 (17.5%)	Post: 11 (20.0%)			
Stage	Beginning: 42 (62.7%)	Beginning: 43 (53.8%)	Beginning: 24 (44.4%)	χ^2^ = 16.58, df = 4, p = 0.002**	V = 0.20	
	Highest: 13 (19.4%)	Highest: 29 (36.3%)	Highest: 28 (51.9%)			
	End: 12 (17.9%)	End: 8 (10.0%)	End: 2 (3.7%)			
Hours/week study	27.56 ± 16.49	29.88 ± 16.62	20.04 ± 14.08	F = 6.22, df = 2, p = 0.002**	η^2^ = 0.06	
Hours/week sport	8.99 ± 5.37	16.38 ± 25.31	19.88 ± 8.52	F = 6.92, df = 2, p = 0.001**	η^2^ = 0.06	

*p < 0.05; **p < 0.01; ***p < 0.001; – no significant differences.

Education: *Pre* pre-university studies, *UD* university degree, *Post* post-university degree.

Disability: *H* hearing, *V* visual, *Ph* physical, *CP* cerebral palsy.

Depending on the level of sports professionalization, significant differences were found in the type of disability (p = 0.007). The highest percentage of amateur athletes had a physical disability or cerebral palsy; the highest percentage of semi-professional athletes had a physical or sensory disability; and the highest percentage of professional athletes had a physical or visual disability. Differences were also found according to the level of professionalization in the stage of their sports career (p = 0.002). Thus, amateur and semi-professional athletes were more likely to be at the beginning of their sporting career, while professional athletes were more likely to be at the peak of their career. Lastly, significant differences were found according to the level of professionalization in the hours per week studying (p = 0.002) and doing sports (p = 0.001). The Bonferroni adjustment showed that, regarding hours spent studying, professional athletes spent significantly fewer hours than semi-professional athletes (Mean dif.: 9.83 ± 2.84; p = 0.002; 95%ICC 2.96; 16.70) and amateur athletes (Mean dif.: 7.51 ± 2.92; p = 0.033; 95%ICC 0.44; 14.59). Regarding the hours spent in their sports, amateur athletes spent significantly fewer hours than professional athletes (Mean dif.: − 10.88 ± 3.04; p = 0.001; 95%ICC − 18.24; − 3.53) and semi-professional athletes (Mean dif.: − 7.39 ± 2.77; p = 0.025; 95%ICC − 14.08; − 0.70).

### Measurements

#### Perceptions of dual career student-athletes

To measure the perceptions of dual career student-athletes, the ‘Perceptions of dual career student-athletes’ (ESTPORT) questionnaire^54^ was used, as in previous research in dual career athletes with and without disability^14,39,55^. The questionnaires were completed in English by all participants. The internal consistency of the questionnaire is high (Cronbach's alpha coefficient > 0.70) with a value of α = 0.857 obtained in the present study, understood as a high reliability^56^. The original version is composed of 84 items. To obtain information about sociodemographic and contextual variables, questions number 1, 2, 7, 8, 9, were included. Furthermore, to know the difficulty of reconciling sporting and academic life, question 20 was also included. Finally, to discover the perceived barriers, the scores obtained in items 26 to 37 of the questionnaire were included. These questions used a Likert scale from 1 (strongly disagree) to 5 points (strongly agree).

#### Exercise benefits/barriers

To analyze the exercise benefits and barriers, the ‘Exercise Benefits/Barriers Scale’ (EBBS)^57^ was used. The questionnaires were completed in English by all participants. The resulting instrument was tested for internal consistency (α = 0.954), validity of its constructs (variance explained: 65.2%), and test–retest reliability (ICC = 0.89)^57^. The Cronbach's alpha coefficient in this study was α = 0.776, understood as a high reliability^56^. This questionnaire has been used in research for the analysis of exercise benefits/barriers in population with disabilities^58^ , athletes^59^ or university students^60^. For this research, items about the barrier scale were included. These questions used a Likert scale from 4 (strongly agree) to 1 (strongly disagree).

#### Athletic identity

The reduced version of the 'Athletic Identity Measurement Scale' (AIMS) was used^61^ to measure athletic identity. The questionnaires were completed in English by all participants. The Cronbach's alpha coefficient in this study was α = 0.776, understood as a high reliability^56^. This questionnaire has been widely used in athletes^62,63^ or dual career students-athletes^64^. The AIMS is composed of seven items designed to assess aspects of athletic identification, with the athlete's role measured on a scale ranging from 1 (strongly disagree) to 7 (strongly agree).

### Procedure

Universities from the participating countries (Spain, Portugal, Italy, Ireland, and Romania) contacted their sports service to send the questionnaire to athletes with disabilities enrolled at the universities, as well as local, regional and national associations, and foundations whose main focus was on athletes with disabilities, and the country's Paralympic Committee. The questionnaire was then circulated by email to all disabled athletes in their databases, specifying that it should only be completed by those who were currently enrolled in pre-university studies, university degree, or university post-degree studies.

The participants completed an informed consent form and a questionnaire anonymously and individually, without academic or competitive pressure. All the questionnaires were completed in English by all participants. The questionnaire was disseminated through Google Forms®, and the participants completed it in 20–30 min. All the data was collected anonymously.

### Statistical analysis

The normality of the data was initially assessed with the Kolmogorov–Smirnov test, homogeneity with the Levene’s test, and sphericity with the Mauchly test. All the variables included in the analysis showed a normal distribution, so parametric tests were performed. The descriptive analysis of quantitative variables shows mean values and standard deviations, while frequencies and percentages were calculated for qualitative variables.

The chi-square analysis (χ^2^) made it possible to establish the differences in the qualitative variables (gender, type of disability, level of education, level of sport professionalism, and stage of the sports career), depending on type of disability and level of sports professionalization. Cramér’s V was used for the post hoc comparison, and the contingency coefficient was used to obtain the statistical value. The maximum expected value was 0.707; r < 0.3 indicated a low association; r < 0.5 indicated a moderate association; and r > 0.5 indicated a high association^65^.

For the analysis of the differences in the quantitative variables (age, hours studying/doing sports, difficulty in reconciling sport and studies, barriers towards achieving a good balance between sport and studies, exercise benefits/barriers and athletic identity) depending on type of disability and level of sports professionalization, a one-way analysis of variance (ANOVA) was used, with the Bonferroni pairwise comparison carried out with the variables with statistical significance, adjusting for the value of p. Partial eta squared (η2) was used to calculate the effect size (ES), and was defined as small: ES ≥ 0.10; moderate: ES ≥ 0.30; large: ES ≥ 1.2; very large: ES ≥ 2.0^66^. The p < 0.05 value was set to determine statistical significance. The statistical analysis was performed using the SPSS statistical package (v.25.0; SPSS Inc., IL, United States). The database of the present project can be found in Zenodo (Zenodo, Netherlands) in open access under https://doi.org/10.5281/zenodo.7956895.

## Results

Table 2 shows the differences in dual career barriers; exercise barriers and athletic identity depending on type of disability. Student-athletes showed statistically significant differences in difficulty in reconciling sports and studies depending on type of disability (p = 0.001), with the Bonferroni adjustment showing that visually impaired student-athletes perceived statistically less difficulty than those with a hearing (Mean dif.: − 0.54 ± 0.19; p = 0.029; 95%ICC − 1.06; − 0.03) or physical (Mean dif.: − 0.59 ± 0.15; p = 0.001; 95%ICC − 1.01; − 0.18) impairment.

**Table 2:** Differences in dual career barriers; exercise barriers and athletic identity depending on type of disability.

		Hearing (n = 34)	Visual (n = 44)	Physical (n = 87)	Cerebral palsy (n = 38)	Group differences (F, df, p)	η^2^
		(Mean ± SD)					
	Difficulty in reconciling sports and studies (scale: 1 to 5)	3.41 ± 0.82	2.86 ± 0.70	3.46 ± 0.09	3.10 ± 0.84	F = 5.64, df = 3, p = 0.001**	0.08
Barriers towards achieving a good balance between sporting life and studies (scale: 1 to 5)	The university/educational institution is far from my home	3.23 ± 1.15	2.90 ± 1.37	3.08 ± 1.39	3.26 ± 1.36	F = 0.596, df = 3, p = 0.618	–
	The university/educational institution is far from my training center	3.17 ± 1.14	3.06 ± 1.40	3.11 ± 1.35	3.34 ± 1.43	F = 0.332, df = 3, p = 0.802	–
	I find myself unable to balance study and training time	2.58 ± 1.37	2.40 ± 1.31	2.77 ± 1.30	2.50 ± 1.35	F = 0.850, df = 3, p = 0.468	–
	My current job does not allow me to study enough	2.96 ± 1.20	2.27 ± 1.34	2.68 ± 1.49	2.42 ± 1.46	F = 1.66, df = 3, p = 0.177	–
	I have to take care of my family	2.44 ± 1.23	1.95 ± 1.19	2.19 ± 1.34	2.34 ± 1.52	F = 1.00, df = 3, p = 0.392	–
	I am usually tired	3.21 ± 1.15	2.75 ± 1.27	3.06 ± 1.28	2.97 ± 1.32	F = 0.93, df = 3, p = 0.427	–
	I lose the rhythm of the academic year	2.91 ± 1.19	2.88 ± 1.22	3.01 ± 1.31	2.63 ± 1.36	F = 0.77, df = 3, p = 0.512	–
	I lose touch with my classmates	3.11 ± 1.34	2.59 ± 1.36	2.77 ± 1.32	2.76 ± 1.40	F = 0.99, df = 3, p = 0.398	–
	The cost of education is high	3.17 ± 1.52	2.25 ± 1.22	2.52 ± 1.37	2.81 ± 1.43	F = 3.26, df = 3, p = 0.023*	0.05
	I do not have enough university/educational institution support	3.08 ± 1.44	2.70 ± 1.21	3.05 ± 1.46	3.68 ± 1.14	F = 3.63, df = 3, p = 0.014*	0.05
	Students’ timetables are not flexible	3.41 ± 1.25	2.95 ± 1.29	3.29 ± 1.43	3.52 ± 1.20	F = 1.42, df = 3, p = 0.238	–
	Training’s timetables are not flexible	2.81 ± 1.25	2.70 ± 1.35	2.84 ± 1.43	3.11 ± 1.34	F = 0.58, df = 3, p = 0.626	–
Exercise benefits/barriers scale (scale: 1 to 4)	Exercising takes too much of my time	1.97 ± 0.83	2.41 ± 0.95	2.24 ± 0.93	2.02 ± 0.75	F = 2.13, df = 3, p = 0.097	–
	Exercise tires me	1.97 ± 0.75	2.52 ± 0.90	2.58 ± 1.00	2.50 ± 0.97	F = 3.66, df = 3, p = 0.013*	0.05
	Places for me to exercise are too far away	2.02 ± 0.79	2.52 ± 0.99	2.17 ± 0.95	2.36 ± 0.97	F = 2.23, df = 3, p = 0.086	–
	I am too embarrassed to exercise	1.35 ± 0.59	1.59 ± 1.01	1.41 ± 0.81	1.23 ± 0.63	F = 1.39, df = 3, p = 0.247	–
	It costs too much to exercise	1.58 ± 0.65	1.88 ± 0.82	1.82 ± 0.91	1.76 ± 0.85	F = 0.88, df = 3, p = 0.448	–
	Exercise facilities do not have convenient timetables for me	2.17 ± 1.05	2.13 ± 1.06	2.56 ± 1.14	3.02 ± 0.94	F = 5.77, df = 3, p = 0.001**	0.08
	I am fatigued by exercise	1.67 ± 0.80	2.00 ± 0.91	2.02 ± 0.97	1.63 ± 0.81	F = 2.48, df = 3, p = 0.062	–
	My spouse (or significant other) does not encourage exercising	1.29 ± 0.62	1.62 ± 0.95	1.34 ± 0.73	1.29 ± 0.57	F = 1.93, df = 3, p = 0.125	–
	Exercise takes too much time from family relationships	2.02 ± 0.86	2.06 ± 0.97	2.06 ± 0.99	1.78 ± 1.06	F = 0.79, df = 3, p = 0.500	–
	I think people in exercise clothes look funny	2.47 ± 1.02	2.54 ± 1.02	2.20 ± 1.12	2.39 ± 1.12	F = 1.13, df = 3, p = 0.336	–
	My family members do not encourage me to exercise	1.47 ± 0.74	1.46 ± 0.79	1.39 ± 0.86	1.42 ± 0.82	F = 0.11, df = 3, p = 0.950	–
	Exercise takes too much time from my family responsibilities	1.73 ± 0.79	1.97 ± 0.97	1.88 ± 0.94	1.84 ± 0.91	F = 0.45, df = 3, p = 0.712	–
	Exercise is hard work for me	1.85 ± 0.78	2.09 ± 0.93	1.97 ± 0.98	2.21 ± 0.93	F = 1.03, df = 3, p = 0.380	–
	There are too few places for me to exercise	1.88 ± 0.94	2.46 ± 1.07	2.19 ± 1.16	2.21 ± 1.16	F = 1.73, df = 3, p = 0.161	–
Athletic identity measurement scale (scale: 1 to 7)	I consider myself an athlete	5.67 ± 1.49	5.70 ± 1.59	5.48 ± 1.63	5.00 ± 1.57	F = 1.61, df = 3, p = 0.187	–
	I have many goals related to sports	5.82 ± 1.48	5.59 ± 1.70	5.65 ± 1.61	5.31 ± 1.78	F = 0.61, df = 3, p = 0.604	–
	Most of my friends are athletes	5.05 ± 1.51	4.50 ± 1.69	4.10 ± 1.81	4.36 ± 1.66	F = 2.60, df = 3, p = 0.053	–
	Sports are the most important part of my life	5.58 ± 1.35	4.97 ± 1.54	5.11 ± 1.81	4.89 ± 1.70	F = 1.22, df = 3, p = 0.301	–
	I spend more time thinking about sports than anything else	4.73 ± 1.48	4.29 ± 1.84	4.75 ± 1.81	4.36 ± 1.90	F = 0.92, df = 3, p = 0.432	–
	I feel bad about myself when I do poorly in sports	4.52 ± 1.63	4.83 ± 1.58	5.10 ± 1.75	4.52 ± 2.07	F = 1.39, df = 3, p = 0.247	–
	I would be very depressed if I were injured and could not compete in sports	5.20 ± 1.70	4.97 ± 1.87	5.35 ± 1.75	5.31 ± 2.00	F = 0.45, df = 3, p = 0.717	–

*p < 0.05; **p < 0.01; ***p < 0.001; – no significant differences.

Statistically significant differences were also found in the perception that ‘the cost of education is high’ (p = 0.023) and ‘I do not have enough university/educational institution support’ (p = 0.014) as a barrier, with student-athletes with a hearing impairment showing higher scores as compared to those with a visual impairment in the first one (Mean dif.: − 0.92 ± 0.31; p = 0.022; 95%ICC − 1.76; − 0.08), and with student-athletes with cerebral palsy showing higher scores as compared to those with a visual impairment in the second (Mean dif.: − 0.97 ± 0.29; p = 0.008; 95%ICC − 1.77; − 0.18).

On the barrier ‘Exercise tires me’ significant differences were also found depending on the type of disability (p = 0.013). The Bonferroni adjustment showed that student-athletes with a physical impairment had significantly higher scores than those with a hearing impairment (Mean dif.: 0.61 ± 0.19; p = 0.009; 95%ICC 1.12; 0.10). On the barrier ‘Exercise facilities do not have convenient timetables for me’ (p = 0.001), participants with cerebral palsy showed statistically higher scores than those with a hearing (Mean dif.: − 0.84 ± 0.25; p = 0.006; 95%ICC − 1.52; − 0.16) and visual (Mean dif.: − 0.88 ± 0.23; p = 0.002; 95%ICC − 1.52; − 0.25) impairment.

With regard to athletic identity according to the disability type, no statistically significant differences were found in the data analyzed.

Table 3 shows the differences in dual career barriers; exercise barriers and athletic identity depending on sports professionalization level. On the student-athletes' reported barriers to dual career success, significant differences were found in the barrier ‘the university/educational institution is far from my training center’ (p = 0.040), with semi-professional athletes showing statistically higher scores than professional athletes (Mean dif.: 0.58 ± 0.23; p = 0.037; 95%ICC 0.02; 1.15). Significant differences were also found in the barrier ‘exercise takes too much time from family responsibilities’ (p = 0.034), with professional athletes showing significantly higher values than amateur athletes (Mean dif.: − 0.45 ± 0.17; p = 0.033; 95%ICC − 0.87; − 0.02).

**Table 3:** Differences in dual career barriers; exercise barriers and athletic identity depending on sports level of professionalization.

		Amateur (n = 68)	Semi-professional (n = 80)	Professional (n = 55)	Group differences (F, df, p)	η^2^
		(Mean ± SD)				
	Difficulty in reconciling sports and studies (scale: 1 to 5)	3.11 ± 0.82	3.32 ± 0.86	3.32 ± 0.92	F = 1.26, df = 2, p = 0.286	–
Barriers towards achieving a good balance between sporting life and studies (scale: 1 to 5)	The university/educational institution is far from my home	2.98 ± 1.22	3.35 ± 1.27	2.89 ± 1.54	F = 2.314, df = 2, p = 0.101	–
	The university/educational institution is far from my training center	3.10 ± 1.23	3.42 ± 1.21	2.83 ± 1.57	F = 3.283, df = 2, p = 0.040*	0.03
	I find myself unable to balance study and training time	2.77 ± 1.23	2.42 ± 1.28	2.67 ± 1.47	F = 1.404, df = 2, p = 0.248	–
	My current job does not allow me to study enough	2.27 ± 1.33	2.72 ± 1.47	2.77 ± 1.36	F = 2.03, df = 2, p = 0.135	–
	I have to take care of my family	2.20 ± 1.32	2.31 ± 1.42	2.10 ± 1.21	F = 0.52, df = 2, p = 0.593	–
	I am usually tired	3.12 ± 1.91	2.93 ± 1.26	2.96 ± 1.38	F = 0.45, df = 2, p = 0.638	–
	I lose the rhythm of the academic year	2.91 ± 1.08	2.91 ± 1.28	2.85 ± 1.50	F = 0.42, df = 2, p = 0.958	–
	I lose touch with my classmates	2.57 ± 1.13	3.02 ± 1.43	2.72 ± 1.44	F = 2.15, df = 2, p = 0.119	–
	The cost of education is high	2.48 ± 1.28	2.72 ± 1.50	2.67 ± 1.40	F = 0.56, df = 2, p = 0.569	–
	I do not have enough university/educational institution support	2.97 ± 1.32	3.26 ± 1.38	3.03 ± 1.43	F = 0.91, df = 2, p = 0.404	–
	Students’ timetables are not flexible	3.26 ± 1.19	3.30 ± 1.34	3.29 ± 1.51	F = 0.13, df = 2, p = 0.987	–
	Training’s timetables are not flexible	3.18 ± 1.18	2.66 ± 1.35	2.77 ± 1.52	F = 2.36, df = 2, p = 0.097	–
Exercise benefits/barriers scale (scale: 1 to 4)	Exercising takes too much of my time	2.26 ± 0.74	2.01 ± 0.83	2.37 ± 1.12	F = 2.91, df = 2, p = 0.056	–
	Exercise tires me	2.60 ± 0.97	2.31 ± 0.89	2.47 ± 1.01	F = 1.70, df = 2, p = 0.184	–
	Places for me to exercise are too far away	2.25 ± 0.90	2.31 ± 1.00	2.20 ± 0.95	F = 0.23, df = 2, p = 0.793	–
	I am too embarrassed to exercise	1.33 ± 0.63	1.46 ± 0.91	1.41 ± 0.83	F = 0.44, df = 2, p = 0.645	–
	It costs too much to exercise	1.80 ± 0.79	1.79 ± 0.83	1.74 ± 0.92	F = 0.09, df = 2, p = 0.910	–
	Exercise facilities do not have convenient timetables for me	2.42 ± 1.11	2.60 ± 1.06	2.41 ± 1.21	F = 0.60, df = 2, p = 0.546	–
	I am fatigued by exercise	1.95 ± 0.96	1.83 ± 0.87	1.87 ± 0.92	F = 0.31, df = 2, p = 0.732	–
	My spouse (or significant other) does not encourage exercising	1.33 ± 0.68	1.38 ± 0.70	1.46 ± 0.88	F = 0.44, df = 2, p = 0.643	–
	Exercise takes too much time from family relationships	1.83 ± 0.92	1.96 ± 0.93	2.29 ± 1.08	F = 3.44, df = 2, p = 0.034*	0.03
	I think people in exercise clothes look funny	2.22 ± 1.04	2.40 ± 1.07	2.47 ± 1.15	F = 0.90, df = 2, p = 0.405	–
	My family members do not encourage me to exercise	1.39 ± 0.79	1.35 ± 0.69	1.56 ± 1.01	F = 1.10, df = 2, p = 0.335	–
	Exercise takes too much time from my family responsibilities	1.80 ± 0.86	1.82 ± 0.88	2.01 ± 1.02	F = 0.90, df = 2, p = 0.405	–
	Exercise is hard work for me	2.04 ± 0.90	1.95 ± 0.88	2.10 ± 1.04	F = 0.49, df = 2, p = 0.613	–
	There are too few places for me to exercise	2.38 ± 1.14	2.23 ± 1.09	1.94 ± 1.09	F = 2.38, df = 2, p = 0.095	–
Athletic identity measurement scale (scale: 1 to 7)	I consider myself an athlete	4.57 ± 1.43	5.60 ± 1.46	6.40 ± 1.39	F = 25.07, df = 2, p < 0.001***	0.20
	I have many goals related to sports	4.77 ± 1.60	5.72 ± 1.52	6.45 ± 1.37	F = 19.06, df = 2, p < 0.001***	0.16
	Most of my friends are athletes	3.95 ± 1.57	4.26 ± 1.68	5.14 ± 1.76	F = 8.10, df = 2, p < 0.001***	0.07
	Sports are the most important part of my life	4.30 ± 1.67	5.12 ± 1.54	6.12 ± 1.27	F = 21.60, df = 2, p < 0.001***	0.18
	I spend more time thinking about sports than anything else	4.01 ± 1.76	4.50 ± 1.71	5.40 ± 1.65	F = 10.06, df = 2, p < 0.001***	0.09
	I feel bad about myself when I do poorly in sports	4.42 ± 1.84	4.94 ± 1.60	5.20 ± 1.83	F = 3.19, df = 2, p = 0.043*	0.03
	I would be very depressed if I were injured and could not compete in sports	5.14 ± 1.78	5.40 ± 1.74	5.12 ± 1.95	F = 0.50, df = 2, p = 0.604	–

*p < 0.05; **p < 0.01; ***p < 0.001; – no significant differences.

On the athletic identity scale, significant differences were found in the dimensions (a) ‘I consider myself an athlete’ (p < 0.001); (b) ‘I have many goals related to sports” (p < 0.001); (c) ‘Most of my friends are athletes’ (p < 0.001); (d) ‘Sports are the most important part of my life’ (p < 0.001); (e) ‘I spend more time thinking about sports than anything else’ (p < 0.001); and (f) ‘I feel bad about myself when I do poorly in sports’ (p = 0.043), where professional athletes showed statistically higher scores than amateur and semi-professional athletes (a) Mean dif.: − 1.82 ± 0.26; p = 0.000; 95%ICC − 2.45; − 1.19 and Mean dif.: − 0.80 ± 0.25; p = 0.005; 95%ICC − 1.40; − 0.1, respectively; (b) Mean dif.: − 1.67 ± 0.27; p < 0.001; 95%ICC − 2.33; − 1.01 and Mean dif.: − 0.72 ± 0.26; p = 0.019; 95%ICC − 1.36; − 0.09, respectively; (c) Mean dif.: − 1.18 ± 0.30; p < 0.001; 95%ICC − 1.92; − 0.45 and Mean dif.: − 0.88 ± 0.29; p = 0.009; 95%ICC − 1.59; − 0.17, respectively; (d) Mean dif.: − 1.81 ± 0.27; p < 0.001; 95%ICC − 2.48; − 1.15 and Mean dif.: − 1.00 ± 0.26; p = 0.001; 95%ICC − 1.64; − 0.35, respectively; (e) Mean dif.: − 1.38 ± 0.31; p = 0.000; 95%ICC − 2.13; − 0.63 and Mean dif.: − 0.90 ± 0.30; p = 0.009; 95%ICC − 1.62; − 0.17, respectively; and (f) Mean dif.: − 0.77 ± 0.31; p = 0.048; 95%ICC − 1.54; − 0.01, with respect to amateurs). Semi-professional athletes also showed statistically significant differences as compared to amateurs in ‘I consider myself an athlete’ (Mean dif.: − 1.02 ± 0.23; p < 0.001; 95%ICC − 1.59; − 0.45); ‘I have many goals related to sports’ (Mean dif.: − 0.94 ± 0.24; p = 0.001; 95%ICC − 1.54; − 0.34) and ‘Sports are the most important part of my life’ (Mean dif.: − 0.81 ± 0.25; p = 0.004; 95%ICC − 1.42; − 0.20).

## Discussion

The first aim of this study was to analyze the differences in dual career interferences and barriers, exercise barriers, and athletic identity depending on type of disability. This is an issue that has not been addressed in previous research, although it could be very relevant, as it appears that student-athletes with disabilities may perceive more barriers against achieving success in their dual career than their non-disabled peers^39^. However, previous research has not compared the difficulties encountered by student-athletes according to the type of disability, which so far prevents us from knowing which groups of disabled people would need a stronger support network to compensate for the barriers they face in the pursuit of dual career success. Moreover, given the multifactorial nature of the barriers they suffer^67^, it would be appropriate to know what type of barriers are affecting each population the most, in order to be able to carry out specific measures aimed at reducing their incidence in a concrete and efficient way.

An important finding of this research is that there were differences in the perception of barriers depending on the type of disability. More specifically, this study found that visually impaired student-athletes perceived the least difficulties in reconciling studies and sports. This could be due to a growing awareness of the need to adapt the education system to maximize learning opportunities for students with visual impairment^68^, as well as the inclusion of administrators, curriculum planners, classmates and families, for a holistic policy for the visually-impaired learners^69^. The scientific literature has found that pedagogical strategies, learning tools, and external support, are the most effective strategies for the inclusion of students with visual impairment^70^. These facilitators coincide with those required for the implementation of an effective dual career model for student-athletes^71^.

At the other end of the spectrum, student-athletes with cerebral palsy showed the highest scores on the barrier ‘I do not have enough university/educational institution support’. Previous studies have found that an inclusive school culture was crucial for students with cerebral palsy, because this group has been classically excluded from the educational system^72^, being sent to separate classes from their peers^73^, which has led to people with cerebral palsy perceiving a lack of institutional and home-based support to achieve success in education^74^. Indeed, in the case of students with cerebral palsy, this perception of dependency led students to perceive that their success in education was substantially impacted by the capacity of adults in the student's life to collaborate with others^72^. Therefore, knowing that student-athletes with disabilities are especially vulnerable in the area of education, it would be necessary to promote measures for the pursuit of success in this population, to create a sufficient support network, thus putting into practice the European regulations that promote the pursuit of equal opportunities for all subjects in the area of education^38,39,52^.

But the perception of high barriers of students with disabilities is not limited to the area of education. Student-athletes with cerebral palsy also showed the highest scores in the barrier ‘Exercise facilities do not have convenient timetables for me’. This barrier has already been pointed out, together with the lack of sports offerings and the scarcity of opportunities, as the main obstacles faced by athletes with cerebral palsy^75^. In addition, for this group, there are other personal and environmental conditioning factors that affect sports practice^75^ . In this sense, it has been found that belonging to a sports club is defined as a successful strategy to increase the participation in sports of these subjects, which also directly increases their athletic identity and their quality-of-life values^76^. This may be due to the fact that the people around them and socialization through sports practice are the main facilitators of sports practice for this group^77^. Furthermore, in the research by Cleary et al.^78^, it was evidenced that physical activity was promoted in this population when academic work and physical activity were understood as equally important priorities at school, a fact that can be considered as the starting point of the benefits expected in this group through the implementation of the dual career model in higher education.

Another relevant finding of this research was that student-athletes with hearing impairment encountered the most barriers related to the cost of education. Previous studies have pointed to the large economic impact of hearing loss on earning power, which may be largely due to the fact that the disability makes it difficult for disabled students to attend university, hindering their ability to find work and to have an average quality of life^79^. In this regard, research by Hogan et al.^80^ highlighted how people with hearing loss were less likely to be in high-skilled jobs, and were overrepresented among the low-income population. In this regard, while generic job skills are readily available in the higher education environment, other skills important for professional development and job search are more difficult for deaf students to acquire^81^. In addition to job opportunities, deaf students encounter a number of barriers in the educational environment that can increase the investment needed to ensure their inclusion, such as the lack of sign language interpreters, which is a key factor in the accessibility of education for the deaf^82,83^.

Finally, physical impairment student-athletes obtained the highest scores in the barrier ‘Exercise tires me’, suggesting that they may have a greater physiological response to exercise than able-bodied athletes^84^. In addition, fatigue is frequent in adults with physical disabilities^85^, which may be accentuated by the use of mobility aids, such as crutches or wheelchairs^86^, as well as by the limitations of transportation for this group, sometimes requiring more time for travel^87^. In this regard, greater fatigue could be related to a lower socio-economic level, due to the high cost of these implements^88^, as well as the increased logistics and costs of adapted transportation, especially affecting team sports^89^.

Regarding the type of disability and athletic identity, no statistically significant differences were detected in the present research, coinciding with the previous results from Pans et al.^90^. Regardless of whether the athlete has a disability or not, as well as the type of disability, he/she has had to give up other roles to prioritize the sporting one, a fact that is especially detected when the athlete reaches an elite competitive level (e.g., Olympic/Paralympic), which leads to a reinforcement of the athletic identity^91^. Therefore, at these levels, there are previous studies that highlight that in adapted sports it is observed that athletes perceive themselves only as athletes and not as people with disabilities who are practicing a sport^92,93^. However, a strong athletic identity in disabled athletes can be an obstacle when facing sporting retirement, negatively affecting employability^92^.

In view of the above results, the hypothesis that there are differences in the perception of barriers to success in dual careers depending on the type of disability can be accepted. On the other hand, although it was not possible to write a clear hypothesis regarding differences in the perception as an athlete, depending on the type of disability, in the present research it was found that there are no differences in the perception as an athlete depending on the type of disability.

A second aim of the present research was to analyze differences in dual career interference and barriers, exercise barriers, and athletic identity, as a function of the level of professionalization of the sport. Differences were found in the perception of barriers by student-athletes according to their level of professionalization. More specifically, semi-professional student-athletes had higher scores in ‘the university/educational institution is far from my training center’. Previous studies have already shown, in non-disabled student-athletes, that semi-professional student-athletes may have higher scores on perceived barriers than their professional counterparts^94^. This could be due to the fact that semi-professional athletes are forced to make a more equal distribution of time between sports and studies, as they find themselves in a situation where they do not know whether to prioritize their sporting or educational career^15,95^. Not in vain, in this study, it was also found that professional athletes spent the most hours on their sport to the detriment of study hours, while semi-professional athletes sought a better balance between these facets. Prior research has also highlighted that athletes with disabilities self-perceive, to a greater extent, the barriers related to the distance from their training center to the educational institution, as compared to their non-disabled peers^39^. In light of the results of the present research, this is an issue that should be especially taken into account in the case of semi-professional student-athletes. Thus, programs where accessible sports facilities are provided within the educational environment^96,97^, or where online attendance at school is allowed^38^, could be a solution for this population.

The present study also found that professional athletes pointed out the highest values in ‘exercise takes too much time from family responsibilities’, showing differences in this item as compared to amateur athletes. This may be due to the fact that professional athletes have employment contracts related to their sporting performance, which oblige them to devote more time to sports, to the detriment of the time they dedicate to their studies^15,98^ or other obligations, such as taking care of the family^15,95^. In this regard, the supports provided by family, friends, or the community, are essential when practicing sports, especially in the beginning, and for some more severe disabilities, throughout the sporting life^99^. The support from a family prepared for this challenge becomes fundamental for the development of the career of the student-athlete with a disability^100^.

Another important finding of this research was that professional athletes showed the highest values in most items related to their identity as athletes, followed by semi-professional athletes and amateur athletes. This suggests that sports play an important role for individuals with disabilities to stay healthy, build social relationships, increase independence, and achieve personal goals^101–103^, which increase together with the level of professionalization as athletes. Previous studies have pointed to a direct relationship between the level of professionalization of able-bodied student-athletes and the importance they grant to their role as athletes, prioritizing it over their status as students^15,98^. However, there have been documented cases of athletes who, after finishing their sporting career, have suffered identity crises that have negatively conditioned their post-sports development^104^. In this way, a greater commitment during the dual career can enrich the athlete's personality and avoid future identity confusion that may hinder their transition to their post-sporting life^105^.

In view of the results of the present research, the hypotheses about the differences in the perception of barriers and athletic identity of student-athletes with disabilities according to the level of professionalization are assumed to be correct.

In the light of the results of this study, student-athletes with disabilities need support measures, management, and policies^106^ that create a network of political and institutional support to integrate their student life into their sports cycle, and to avoid difficult transitions to work after the end of the sporting career^107,37^. These must be adapted to their perceived barriers and generate specific measures depending on the type of disability and level of sports. However, as this is a pioneer study about dual career of athletes with disabilities, there are potential limitations, such as the heterogeneity of the sample, disabilities, career stages, sport/education careers, age, inclusion of athletes with disabilities independently from their acquired or congenital disabilities, and different models between para-sports organizations and educational institutions/public and private companies in the European Member States. With regard to future lines of research, it would be interesting to analyze whether factors such as gender, the stage of their sporting career, and level of education, could affect the self-perception of dual career barriers and athletic identity of student-athletes with disabilities.

## Conclusion

In conclusion, among student-athletes with disabilities, student-athletes with cerebral palsy, hearing impairment or physical disability, and semi-professional or professional athletes, showed higher scores in the perception of barriers to the success of their dual career. Furthermore, the importance granted by student-athletes with disabilities to their identity as an athlete is higher for professional athletes.

## Data Availability

The database was included in the Zenodo repository (link: https://zenodo.org/record/7956896#.ZGtReS8lOUk).

## References

[1] Vidal-Vilaplana A (2022). Combining sport and academic career: Exploring the current state of student-athletes’ dual career research field. J. Hosp. Leis Sport Tour Educ..

[2] Kristiansen E (2017). Walking the line: How young athletes balance academic studies and sport in international competition. Sport Soc..

[3] Bastianon S, Greco G (2018). The Italian approach to the dual careers of University student-athletes. Kinesiologia Slovenica.

[4] Capranica L (2022). Understanding dual career views of European university athletes: The more than gold project focus groups. PLoS ONE.

[5] Gouttebarge V, Aoki H, Kerkhoffs GM (2016). Prevalence and determinants of symptoms related to mental disorders in retired male professional footballers. J. Sports Med. Phys. Fitness.

[6] Torregrosa M, Ramis Y, Pallarés S, Azócar F, Selva C (2015). Olympic athletes back to retirement: A qualitative longitudinal study. Psychol. Sport Exerc..

[7] Barker D, Barker-Ruchti N, Rynne S, Lee J (2014). Moving out of sports: A sociocultural examination of Olympic career transitions. Int. J. Sports Sci. Coach..

[8] van Rens FECA, Ashley RA, Steele AR (2019). Well-being and performance in dual careers: The role of academic and athletic identities. Sport Psychol..

[9] Moreno-Pérez V (2020). Acute and chronic effects of competition on ankle dorsiflexion ROM in professional football players. Eur. J. Sport Sci..

[10] Vilanova A, Puig N (2017). Estrategias de entrada al mercado de trabajo de los atletas olímpicos. Una tipología. Rev. Int. Sociol..

[11] Isidori E, Fazio A, Angelillo E, Laterza E, Colitti L, Sánchez-Pato A, Isidori E, Calderón A, Brunton J (2017). An innovative European sports tutorship model of the dual career of student-athletes. An Innovative European Sports Tutorship Model of the Dual Career of Student-Athletes.

[12] European Commission. *EU Guidelines on Dual Careers of Athletes Recommended Policy Actions in Support of Dual Careers in High-Performance Sport*. https://ec.europa.eu/assets/eac/sport/library/documents/dual-career-guidelines-final_en.pdf (2012).

[13] Aquilina D (2013). A study of the relationship between elite athletes’ educational development and sporting performance. Int. J. Hist. Sports..

[14] Abenza-Cano L (2020). Effect of Coronavirus Disease 2019 (COVID-19) on Elite Spanish Student-Athletes’ perception of the dual career. Front. Psychol..

[15] Mateo-Orcajada A (2022). Spanish Pre-olympic athletes’ motivations and barriers to pursuing dual career as a function of sociodemographic, sport and academic variables. Front Psychol..

[16] de Subijana CL, Ramos J, Garcia C, Chamorro JL (2020). The employability process of spanish retired elite athletes: Gender and sport success comparison. Int. J. Environ. Res. Public Health.

[17] López de Subijana Hernández C, Barriopedro Moro M, Alberto Muniesa C (2018). La retirada deportiva en deportes colectivos: comparativa profesionales y amateurs. SPORT TK-Revista EuroAmericana de Ciencias del Deporte.

[18] Cosh S, Tully PJ (2014). “All I have to do is pass”: A discursive analysis of student athletes’ talk about prioritising sport to the detriment of education to overcome stressors encountered in combining elite sport and tertiary education. Psychol. Sport Exerc..

[19] Osborne B, Jensen JA, Weight EA (2020). Intercollegiate athletics: A unique segment of the sport industry. J. Glob. Sport Manag..

[20] Houlihan B, Zheng J (2013). The Olympics and elite sport policy: Where Will it all end?. Int. J. Hist. Sport.

[21] Guidotti F, Cortis C, Capranica L (2015). Dual career of European student-athletes: A systematic literature review. Kinesiologia Slovenica.

[22] Migliorati M, Maulini C, Isidori EL (2016). dual-career degli studenti-atleti nella scuola secondaria: fra teoresi pedagogica e progettualità. Rivista internazionale di Scienze dell’educazione e della formazione.

[23] Guidotti F (2013). Validation of the Italian version of the student athletes’ motivation toward sport and academics questionnaire. Sport. Sci. Health.

[24] Lupo C (2015). Motivation towards dual career of European student-athletes. Eur J Sport Sci.

[25] Stambulova N, Schinke RJ, McGannon K, Smith B (2016). Athletes’ transitions in sport and life: Positioning new research trends within the existing system of athlete career knowledge. The Routledge International Handbook of Sport Psychology.

[26] Alfermann, D. & Stambulova, N. Career transitions and career termination. *Handbook of Sport Psychology: Third Edition* 712–733 (Wiley, 2012) 10.1002/9781118270011.CH32.

[27] Stambulova NB, Wylleman P (2019). Psychology of athletes’ dual careers: A state-of-the-art critical review of the European discourse. Psychol. Sport Exerc..

[28] Stambulova NB, Engström C, Franck A, Linnér L, Lindahl K (2015). Searching for an optimal balance: Dual career experiences of Swedish adolescent athletes. Psychol. Sport Exerc..

[29] Moreno R, de Subijana CL, Chamorro JL (2020). “I Never Thought I´d Drop out of School”. The influence of parents academic history in the development of dual career in the elite athletes. Rev. Psicol. Deporte (J. Sport Psychol.).

[30] López de Subijana C, Ramos J, Keith C, Lupo C (2020). Life skills from sport: the former elite athlete’s perception Life skills from sport: the former elite athlete’s perception. Sport Soc..

[31] Lally PS, Kerr GA (2005). The career planning, athletic identity, and student role identity of intercollegiate student athletes. Res. Q. Exerc. Sport.

[32] Klasen, S. A. *An Examination of the Athletic Identity, Identity Foreclosure, and Career Maturity of Division I Collegiate Student-Athletes in Nonrevenue-Producing Sports* (Northern Illinois University, 2016).

[33] Kerstajn R, Lupo C, Capranica L, Topic MD (2018). Motivation towards sports and academics careers in elite winter sport Slovenian and Italian athletes: The role of internal and external factors. Ido Mov. Cult..

[34] Quinaud RT, Capranica L, Doupona M, Guidotti F (2022). The holistic development of talented sportspersons through dual-career. Front. Sports Act Living.

[35] Capranica L (2021). The Contribution of the European Athlete as Student Network (EAS) to European Dual Career ERASMUS+ Sport Collaborative Partnerships: An update (La Contribución de la Red European Athlete as Student (EAS) a las European Dual career ERASMUS + Sport: Una actualización). Cultura, Ciencia y Deporte.

[36] Fuchs PX (2021). Multi-national perceptions on challenges, opportunities, and support structures for Dual Career migrations in European studentathletes. PLoS ONE.

[37] Izzicupo P (2022). Exploring dual career quality implementation at European higher education institutions: Insights from university experts. PLoS ONE.

[38] Magnanini A, Isidori E, Fazio A, Cioni L (2022). The university’s role in the dual career of student-athletes with disabilities: The preliminary data of the “PARA-LIMITS” Project. Giornale Italiano di Educazione alla Salute, Sport e Didattica Inclusiva.

[39] Vaquero-Cristóbal R (2023). Exploring the perception of barriers to a dual career by student-athletes with/out disabilities. PLoS ONE.

[40] López-Flores M, Penado M, Avelar-Rosa B, Packevičiūtė A, Ābeļkalns I (2021). May the Mentor be with You! An innovative approach to the Dual Career mentoring capacitation. Cult. Cienc. Deporte.

[41] Council of Europe. Rights of Persons with Disabilities. https://www.coe.int/en/web/disability (2023).

[42] Duarte T, Culver DM, Paquette K (2020). Mapping Canadian wheelchair curling coaches’ development: A landscape metaphor for a systems approach. Int. Sport Coach J..

[43] European Commission. *Mapping on access to sport for people with disabilities. A Report to the European Commission*. 10.2766/061635 (2018).

[44] Patatas JM, De Bosscher V, Legg D (2018). Understanding parasport: An analysis of the differences between able-bodied and parasport from a sport policy perspective. Int. J. Sport Policy Polit..

[45] Felix-Mena A, Martínez-Rodríguez A, Reche-García C (2021). Resiliencia y burnout en la carrera dual (Resilience and burnout in dual career). Cult. Cienc. Deporte.

[46] Sorkkila M, Ryba TV, Aunola K, Selänne H, Salmela-Aro K (2020). Sport burnout inventory–Dual career form for student-athletes: Assessing validity and reliability in a Finnish sample of adolescent athletes. J. Sport Health Sci..

[47] Pallarés S, Azócar F, Torregrosa M, Selva C, Ramis Y (2011). Modelos de trayectoria deportiva en waterpolo y su implicación en la transición hacia una carrera profesional alternativa (Athletic Career Models in Water Polo and their Involvement in the Transition to an Alternative Career). Cult. Cienc. Deporte.

[48] Sayvon F, Huml M (2017). The relationship between athletic identity and academic major chosen by student-athletes. Int. J. Exerc. Sci..

[49] Simons HD, Van Rheenen D (2000). Noncognitive predictors of student athletes’ academic performance. J. Coll. Read. Learn..

[50] Dipeolu A, Reardon R, Sampson J, Burkhead J (2002). The relationship between dysfunctional career thoughts and adjustment to disability in college students with learning disabilities. J. Career Assess..

[51] Lindsay S, Cagliostro E, Leck J, Stinson J (2021). Career aspirations and workplace expectations among youth with physical disabilities. Disabil. Rehabil..

[52] Madriaga M, Hanson K, Kay H, Walker A (2011). Marking-out normalcy and disability in higher education. Br. J. Sociol. Educ..

[53] Vandenbroucke JP (2014). Strengthening the Reporting of Observational Studies in Epidemiology (STROBE): Explanation and elaboration. Int. J. Surg..

[54] Sánchez-Pato A (2016). Design and validation of a questionnaire about the perceptions of dual career student-athletes (ESTPORT). Cult. Cienc. Deporte.

[55] Gavala-González J, Castillo-Rodríguez A, Fernández-García JC (2019). Dual career of the U-23 Spanish canoeing team. Front. Psychol..

[56] Corbetta, P. *Social Research Methodologies and Techniques* (McGraw, 2007).

[57] Sechrist KR, Walker SN, Pender NJ (1987). Development and psychometric evaluation of the exercise benefits/barriers scale. Res. Nurs. Health.

[58] Malone L, Barfield J, Brasher J (2012). Perceived benefits and barriers to exercise among persons with physical disabilities or chronic health conditions within action or maintenance stages of exercise. Disabil. Health J..

[59] Malchrowicz-Mośko E, Wieliński D, Adamczewska K (2020). Perceived benefits for mental and physical health and barriers to horseback riding participation. The analysis among professional and amateur athletes. Int. J. Environ. Res. Public Health.

[60] Arzu D, Tuzun EH, Eker L (2006). Perceived barriers to physical activity in university students. J. Sports Sci. Med..

[61] Brewer B, Cornelius A (2001). Norms and factorial invariance of the Athletic Identity Measurement Scale. Acad. Athletic J..

[62] Lochbaum M, Cooper S, Limp S (2022). The athletic identity measurement scale: A systematic review with meta-analysis from 1993 to 2021. Eur. J. Investig. Health Psychol. Educ..

[63] Edison BR, Christino MA, Rizzone KH (2021). Athletic identity in youth athletes: A systematic review of the literature. Int. J. Environ. Res. Public Health.

[64] Park S, Hong S, Lee M (2015). Validation of the student athletes’ motivation towards sports and academics questionnaire to Korean student-athletes. J. Exerc. Rehabil..

[65] Cramér, H. *Mathematical Methods of Statistics* (Princeton University Press, 1946).

[66] Hopkins WG, Marshall SW, Batterham AM, Hanin J (2009). Progressive statistics for studies in sports medicine and exercise science. Med. Sci. Sports Exerc..

[67] López de Subijana C, Barriopedro M, Conde E (2015). Supporting dual career in Spain: Elite athletes’ barriers to study. Psychol. Sport Exerc..

[68] Reina R, Ruiz JÁ (2016). Full inclusion of a student with visual impairment over the full Physical Activity and Sport Sciences Degree: A case study. Eur. J. Adapt. Phys. Activity.

[69] Kocyigit N, Artar PS (2015). A challenge: Teaching English to visually-impaired learners. Procedia Soc. Behav. Sci..

[70] Miyauchi H (2020). A systematic review on inclusive education of students with visual impairment. Educ. Sci..

[71] Storm LK (2021). Ten essential features of European dual career development environments: A multiple case study. Psychol. Sport Exerc..

[72] Bourke-Taylor HM, Cotter C, Lalor A, Johnson L (2018). School success and participation for students with cerebral palsy: A qualitative study exploring multiple perspectives. Disabil. Rehabil..

[73] Carlon SL, Taylor NF, Dodd KJ, Shields N (2013). Differences in habitual physical activity levels of young people with cerebral palsy and their typically developing peers: A systematic review. Disabil. Rehabil..

[74] Odhiambo JA (2018). Support services available for kenyan learners with cerebral palsy in aid of the performance of activities of daily living. Am. J. Educ. Learn..

[75] Verschuren O, Wiart L, Hermans D, Ketelaar M (2012). Identification of facilitators and barriers to physical activity in children and adolescents with cerebral palsy. J. Pediatr..

[76] Groff DG, Lundberg NR, Zabriskie RB (2009). Influence of adapted sport on quality of life: Perceptions of athletes with cerebral palsy. Disabil. Rehabil..

[77] Rodríguez Macías M, Giménez Fuentes-Guerra FJ, Abad Robles MT (2022). The sport training process of para-athletes: A systematic review. Int. J. Environ. Res. Public Health.

[78] Cleary SL, Taylor NF, Dodd KJ, Shields N (2019). Barriers to and facilitators of physical activity for children with cerebral palsy in special education. Dev. Med. Child Neurol..

[79] Jones DD (2004). Relative earnings of deaf and hard-of-hearing individuals. J. Deaf Stud. Deaf Educ..

[80] Hogan A, O’Loughlin K, Davis A, Kendig H (2009). Hearing loss and paid employment: Australian population survey findings. Int. J. Audiol..

[81] Conway J (2019). The Journal of Inclusive Practice in Further and Higher Education.

[82] Melero C, Hernández Fernández A, Camargo C (2020). Diversidad funcional auditiva en el aula de educación física. Publicaciones.

[83] Hendry G, Hendry A, Ige H, McGrath N (2021). “I was isolated and this was difficult”: Investigating the communication barriers to inclusive further/higher education for deaf Scottish students. Deaf. Educ. Int..

[84] Simim MAM (2018). The demands of amputee soccer impair muscular endurance and power indices but not match physical performance. Adapt. Phys. Activity Q..

[85] Miró J (2022). The Silhouettes Fatigue Scale: A validity study with individuals with physical disabilities and chronic pain. Disabil. Rehabil..

[86] Tatar Y (2018). Load distribution on the foot and lofstrand crutches of amputee football players. Gait Posture.

[87] Arnold R, Wagstaff CRD, Steadman L, Pratt Y (2017). The organisational stressors encountered by athletes with a disability. J Sports Sci.

[88] Buts C, Bois CDu, Heyndels B, Jegers M (2011). Socioeconomic determinants of success at the summer paralympics. J. Sports Econom.

[89] Reina-Vaillo R, Martínez Donoso JL, Jofre Bernardo A, Pérez Bueno LC (2018). The ecosystem of sport for people with disabilities in Spain. White Paper on Sport for People with Disabilities in Spain.

[90] Pans M, Úbeda-Colomer J, Devís-Devís J (2021). Validación de la Athletic Identity Measurement Scale en Estudiantes Universitarios con Discapacidad y Diferencias según Variables Sociodemográficas. Rev. Psicol. Deporte (J. Sport Psychol.).

[91] Ronkainen NJ, Kavoura A, Ryba TV (2016). Narrative and discursive perspectives on athletic identity: Past, present, and future. Psychol. Sport Exerc..

[92] Pack S, Kelly S, Arvinen-Barrow M (2017). “I think I became a swimmer rather than just someone with a disability swimming up and down:” Paralympic athletes perceptions of self and identity development. Disabil. Rehabil..

[93] Guerrero M, Martin J (2018). Para sport athletic identity from competition to retirement. Phys. Med. Rehabil. Clin. N. Am..

[94] Leiva-Arcas, A., Comyns, T. & Ege, H. *European Handbook ‘Para-Limits, Dual Career, Disability and Sport’*. (UCAM Servicio de Publicaciones, 2023).

[95] López de Subijana Hernández C, Barriopedro Moro M, Alberto Muniesa C (2018). La retirada deportiva en deportes colectivos: comparativa profesionales y amateurs. SPORT TK-Rev. EuroAm. Ciencias Deporte.

[96] Jaarsma EA, Dijkstra PU, Geertzen JHB, Dekker R (2014). Barriers to and facilitators of sports participation for people with physical disabilities: A systematic review. Scand. J. Med. Sci. Sports.

[97] Whittingham J, Barker JB, Slater MJ, Arnold R (2021). An exploration of the organisational stressors encountered by international disability footballers. J. Sports Sci..

[98] Gómez M-Á, Lago C, Gómez M-T, Furley P (2019). Analysis of elite soccer players’ performance before and after signing a new contract. PLoS ONE.

[99] Mendoza Laiz N, Sanz Rivas D, Reina Vaillo R, Martínez Donoso JL, Jofre Bernardo A, Pérez Bueno LC (2018). People with disabilities and sport in Spain. General introduction. White Paper on Sport for People with Disabilities in Spain.

[100] Dehghansai N, Pinder RA, Baker J, Renshaw I (2021). Challenges and stresses experienced by athletes and coaches leading up to the Paralympic Games. PLoS ONE.

[101] Lumsdaine G, Lord R (2021). (Re)creating a healthy self in and through disability sport: autoethnographic chaos and quest stories from a sportswoman with cerebral palsy. Disabil. Soc..

[102] Wiesel I, Bigby C, van Holstein E, Gleeson B (2022). Three modes of inclusion of people with intellectual disability in mainstream services: mainstreaming, differentiation and individualisation. Disabil. Soc..

[103] Comella A, Hassett L, Hunter K, Cole J, Sherrington C (2019). Sporting opportunities for people with physical disabilities: Mixed methods study of web-based searches and sport provider interviews. Health Promot. J. Aust..

[104] Lally P (2007). Identity and athletic retirement: A prospective study. Psychol. Sport Exerc..

[105] Park S, Lavallee D, Tod D (2013). Athletes’ career transition out of sport: A systematic review. Int. Rev. Sport Exerc. Psychol..

[106] Patatas JM, De Bosscher V, Derom I, De Rycke J (2020). Managing parasport: An investigation of sport policy factors and stakeholders influencing para-athletes’ career pathways. Sport Manag. Rev..

[107] Izzicupo P (2021). Dual careers of athletes during COVID-19 lockdown. Front. Psychol..

